# Supplementary material for: Genetic and environmental influences on the distributions of three chromosomal drive haplotypes in maize
Source: bioRxiv. 2025 May 27:2025.05.22.655462. Preprint. [Version 1] doi: 10.1101/2025.05.22.655462 (PMC12154789; doi:10.1101/2025.05.22.655462)
Supplement: Supplement 1 [file NIHPP2025.05.22.655462v1-supplement-1.pdf]

bioRxiv preprint doi: https://doi.org/10.1101/2025.05.22.655462; this version posted May 27, 2025. The copyright holder for this preprint (which was not certified by peer review) is the author/funder, who has granted bioRxiv a license to display the preprint in perpetuity. It is made available under aCC-BY 4.0 International license.

Table S1. Summary table of all CDHs identified.

Table S2. Inbred lines that scored positive for CDHs, and PCR data from a subset.

Table S3. All CDH associated SNPs and Genes they overlap.

Table S1. Accessions and individuals assayed, and lines that show both B chromosome and Ab10 or B chromosome and K10L2.

Table S5. Primers used for genotyping.

Figure S1. Workflow diagrams. **A**. Workflow diagram for the generation of the tag index for CDHs and single copy core genes. **B**. Diagram of the workflow for detecting CDHs in experimental samples. Check indicates passing, x indicates failing.

Figure S2. Identification of Ab10 type. **A.** Min/Max scaled tag index for all Ab10 positive control

bioRxiv preprint doi: https://doi.org/10.1101/2025.05.22.655462; this version posted May 27, 2025. The copyright holder for this preprint (which was not certified by peer review) is the author/funder, who has granted bioRxiv a license to display the preprint in perpetuity. It is made available under aCC-BY 4.0 International license.

and experimental samples. Ab10 types classified by the random forest model are plotted separately. Each group is ward.D clustered. The x axis shows individual samples, the y axis shows features of the Ab10 haplotype and the importance of each 1 Mb bin in determining Ab10 type in the random forest model (mean decreasing Gini). The RF confidence value indicates the proportion of decision trees that are called the predominant Ab10 type. **B**. A PCA of all the Ab10 positive samples scaled tag index with controls and their type indicated.

Figure S3. Location of SNPs used for GWAS. Numbers below the CDH name indicate the total number of SNPs.

Figure S4. Manhattan plots of SNPs that passed through filtering but lie within or close to a CDH. Numbers below the CDH names indicate the number of SNPs in the plot. Dotted grey line indicates a p value of $5x10^{-8}$.

Figure S5. Relationship of CDHs to elevation. N.S. indicates not significant.

Figure S6. Plots of simplified generalized linear models for each CDH including population structure and environmental variables (but not genetic modifiers). Shape color and orientation indicate the direction of the relationship to the CDH. Shape size represents the effect size.

Figure S7. Correlation matrix for selected environmental variables.
